# No difference in strength and clinical outcome between early and late repair after Achilles tendon rupture

**DOI:** 10.1007/s00167-018-5340-5

**Published:** 2018-12-29

**Authors:** Michael R. Carmont, Jennifer A. Zellers, Annelie Brorsson, Karin Grävare Silbernagel, Jón Karlsson, Katarina Nilsson-Helander

**Affiliations:** 1 Department of Orthopaedic Surgery, Princess Royal Hospital, Shrewsbury and Telford Hospital NHS Trust, Shropshire, UK; 2 Department of Orthopaedic Surgery, Sahlgrenska Academy, Gothenburg University, Gothenburg, Sweden; 3 Program of Physical Therapy, Washington University School of Medicine, St Louis, MO USA; 4 Department of Physical Therapy, University of Delaware, Newark, DE USA

## Abstract

**Purpose:**

This retrospective study aimed to determine the patient-reported and functional outcome of patients with delayed presentation, who had received no treatment until 14 days following injury of Achilles tendon rupture repaired with minimally invasive surgery and were compared with a group of sex- and age-matched patients presenting acutely. Based on the outcomes following delayed presentation reported in the literature, it was hypothesized that outcomes would be inferior for self-reported outcome, tendon elongation, heel-rise performance, ability to return to play, and complication rates than for acutely managed patients.

**Methods:**

Repair was performed through an incision large enough to permit mobilisation of the tendon ends, core suture repair consisting of a modified Bunnell suture proximally and a Kessler suture distally and circumferential running suture augmentation.

**Results:**

Nine patients presented 21.8 (14.9) days (range 14–42 days) after rupture. The rate of delayed presentation was estimated to be 1 in 10. At 12 months following repair, patients with delayed treatment had median (range) ATRS score of 90 (69–99) compared with 94 (75–100) in patients treated acutely presenting 0.66 (1.7) (0–5) days. There were no significant differences between groups: ATRA [mean (SD) delayed: − 6.9° (5.5), acute: − 6° (4.7)], heel-rise height index [delayed: 79% (20), acute: 74% (14)], or heel-rise repetition index [delayed: 77% (20), acute: 71% (20)]. In the delayed presentation group, two patients had wound infection and one iatrogenic sural nerve injury.

**Conclusions:**

Patients presenting more than 2 weeks after Achilles tendon rupture may be successfully treated with minimally invasive repair.

**Level of evidence:**

III.

**Electronic supplementary material:**

The online version of this article (10.1007/s00167-018-5340-5) contains supplementary material, which is available to authorized users.

## Introduction

Late presentation and diagnosis of Achilles tendon ruptures occurs in as many as 1 in 5 patients, with symptomatic patients reporting an abnormal gait with an inability to push off and persistent weakness [17, 21]. This may be due to a lack of appreciation of the injury or an inaccurate history resulting in delayed diagnosis. Once the diagnosis of Achilles tendon rupture is made, the aim of initial treatment is to appose tendon ends. Ultimately, the goal is to restore function including ankle range of movement and plantar flexion strength whilst minimising complications.

There is continued debate whether operative or non-operative treatment is to be preferred after acute Achilles tendon rupture [24, 25, 29]. However, in the context of delayed treatment, there is concern for poor long-term prognosis if apposition of the ruptured tendon ends has not been achieved within 2 weeks of the injury. In this case, operative repair is generally recommended [9, 16] to mobilise separated tendon ends and/or reconstruct an absent tendon to minimise gap formation, non-healing and resultant dysfunction [22].

Open repair is considered the standard technique for the repair of acute ruptures [24, 25], but there is increasing evidence for lower infection rates and wound breakdown with minimally invasive repair [31]. In case of chronic rupture, usually defined as initiation of treatment more than 6 weeks following injury, open repair either involves the use of an extensive open incision to permit V–Y plasty, fascial graft augmentation [23] or hamstring reconstruction with a wound complication rate of almost 25% [21]. For chronic ruptures, minimally invasive reconstruction rather than repair is increasingly used, although this introduces the risk of complications associated with autograft and allograft use. There are only a few studies on the outcome of repair in the acute-on-chronic time period following delayed presentation [1, 2, 23].

Although the use of minimally invasive repair techniques for patients presenting after the acute phase following injury presents a potentially attractive option, only one series has been reported. Anathatee et al. performed end-to-end repair using the Achillon jig (Integra, Plainsboro, NJ, USA) forming a box suture, at 11–31 days following rupture [1]. Percutaneous suture configurations, including the box suture, are weaker than open configurations on biomechanical testing [8, 30] resulting in increased ankle dorsiflexion [15]. Good clinical outcomes are reported, using modified Bunnell and Kessler configurations following acute repair [4–6]. The use of a minimally invasive repair permits the augmentation of the core suture with a peripheral epitenon running suture to increase repair strength [14, 20].

This study aimed to evaluate the recovery of symptoms/disability and functional outcome of patients after Achilles tendon repair, with minimally invasive surgery including peripheral circumferential running suture, in patients with delayed presentation compared with patients treated acutely post rupture. Based on the outcomes following delayed presentation reported in the literature, it was hypothesized that outcomes of patients with delayed presentation would be inferior for self-reported outcome, tendon elongation, heel-rise performance, ability to return to play, and complication rates than those for acutely managed patients.

## Materials and methods

The outcome of patients presenting with delayed presentation following Achilles tendon rupture to Princess Royal Hospital, Shropshire, United Kingdom, a District General Hospital, between 2014 and 2017 was assessed. Patients were included in the delayed presentation group if they presented after 14 days following injury and had received no treatment during this time period. The comparison group consisted of an equal number of matched patients who had presented, underwent treatment and had acute repair within 14 days of injury (Table 1). The comparison patients were selected retrospectively but had received treatment during the same time period. Patients were matched according to sex and to the nearest possible age. The first patient of comparable age was chosen to minimise potential bias. Five years of difference was chosen for the upper limit of age matching based on prior literature suggesting changes in outcome with age differences greater than 10 years [26]. There was no comparison patient within 5 years of age and of the same sex for one of the patients with delayed presentation. In this case, an appropriately aged patient of the opposite sex was included.

**Table 1:** Demographic details of the delayed presentation and acute control groups

Mean (SD) Median (range)	Delayed presentation	Acute control	*p* value
Number (*n*=)	9	9	
Elapsed time to treatment/days	21.8 (8.5) (14–42)	0.66 (1.7) (0–5)	< 0.001*
Elapsed time from commencement of treatment to repair/days	3.4 (2.7) (0–9)	4.44 (2.6) (0–8)	n.s.
Age/years	48.4 (14.9)	47.7 (14.6)	n.s.
Male:female ratio	8:1	9:0	< 0.001*
Weight/kg	89.2 (16.2)	98.6 (20.7)	n.s.
Body mass index	29.6 (5.5)	30.2 (5.3)	n.s.
Pre-injury Tegner	6.2 (1.9), 7 (3–9)	6.2 (1.7), 7 (4–9)	n.s.

*Significant value,* n.s.* non-significant

The diagnosis of rupture was made on clinical grounds based upon the history of a pop localised by the patient to the Achilles tendon with subsequent symptomatic lack of plantar flexion strength. The diagnostic signs of mid-substance rupture were a palpable gap to the tendon, an abnormal calf squeeze test and an increased Achilles Tendon Resting Angle (ATRA) compared with the non-injured ankle [5]. Imaging was not used to confirm the diagnosis.

### Surgical technique

Following presentation, patients were placed into a functional brace consisting of a reinforced synthetic cast in plantar flexion and underwent operative repair as soon as theatre time allowed. All repairs were performed by the same surgeon.

In the delayed presentation group, patients were placed in the lateral recovery position, prophylactic flucloxacillin antibiotics and an intermittent calf compression device were used. A longitudinal incision was made on the medial border of the Achilles tendon, was commenced 2 cm proximal to the palpable proximal tendon end and extended as required. A blunt instrument, e.g., a Cobb dissector or a malleable aluminium strip was used to separate the ruptured tendon end from the thickened paratenon layer [18].

**Fig. 1:**
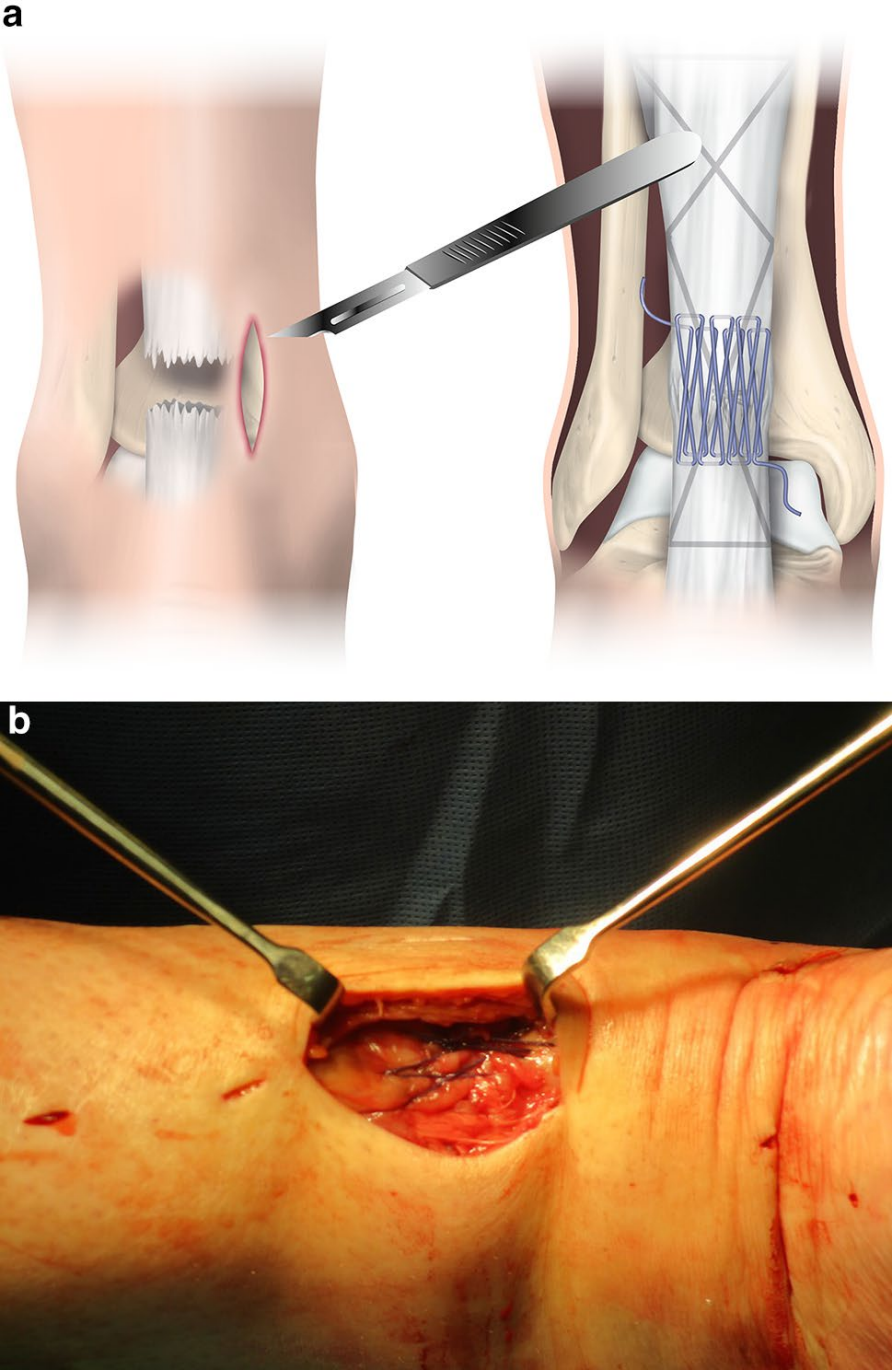
**a, b** The minimally invasive repair suture configuration consisted of a modified Bunnell suture proximally and a Kessler suture distally of Number 2 non-absorbable braided suture. This core suture was augmented by a Silfverskiöld peripheral running suture of Number 0 absorbable braided suture

A 2 cm incision was then made lateral to the tendon 3 cm proximal to the proximal tendon stump and the sural nerve identified, mobilised and protected. Additional five stab incisions were then made as in an established technique [4]. A six strand 2 Fiberwire (Arthrex, Munich, Germany) repair using a core suture consisted of a modified Bunnell suture proximally and a Kessler suture distally. The sutures were tied with the ankle held in full plantar flexion and tied as tightly as possible using a surgeon’s knot and four subsequent throws. With careful retraction, a Silfverskiöld peripheral running suture [25] was applied using a 0 Novosyn (B-Braun, Hessen, Germany) absorbable suture of the accessible tendon ends (Fig. 1a, b). The paratenon and fascia cruris were then carefully closed.

In the acute group, a minimally invasive repair was performed, using an identical core suture and suture configuration, antibiotic prophylaxis and nerve exposure, through a 2–2.5 cm incision at the level of the palpable proximal tendon end [4].

### Post-operative management and rehabilitation

Identical post-operative rehabilitation was undertaken by both the delayed presentation and acute groups. Immediately following operative repair, patients were placed in a functional brace and permitted to mobilise full weight bearing on their metatarsal heads using axillary crutches. Low-molecular weight heparin thromboprophylaxis was used for 6 weeks. At 2 weeks, patients had their skin sutures removed, commenced active plantar flexion, inversion and eversion exercises and maintained the anterior shell held in place for full weight bearing. At 6 weeks, the anterior shell was discontinued, a 1.5 cm heel wedge was provided for full weight bearing until 3 months, and patients were referred for formal strengthening physiotherapy. Patients used crutches until the 8-week time point or when they felt able to resume normal walking. Plyometric exercises were permitted at 3 months. No other restrictions were made in terms of return to activity or sports.

### Outcome evaluation

Patients were evaluated at presentation, immediately following operative repair and at 6 weeks, 3, 6, 9 and 12 months using the relative ATRA as a clinical measure of approximation of tendon ends [4] and tendon elongation (*ρ* = 0.491, *p* = 0.001) [32] and calf circumference [4]. The ATRA was measured with the patient prone and the knee flexed to 90°. The ATRA is the angle between the long axis of the fibula and the line from the tip of the fibula to the head of the fifth metatarsal [5]. A goniometer with 1° graduation and 30 cm long arms was used (66 fit Limited, Spalding, UK) [6]. The relative ATRA is the difference between the injured and non-injured sides. The calf circumference was measured using a standard tape measure with 1 mm increments. The patient’s Achilles tendon Total Rupture Score, Tegner, Halasi and Physical Activity Scores were determined at each follow-up visit from 3 months [6], together with the patients perception of performance (PPP) [6]. Other physical parameters included the Heel-Rise Height Index (HRHI) [6] and Heel-Rise Repetition Index (HRRI) from 3 months to assess functional rehabilitation strength. The term index is used as a comparison of the affected and non-affected side (injured/uninjured × 100 given in %). The HRRI was evaluated by counting the number of single heel rises made until fatigued. The injured ankle was tested first. Patients were permitted to place their fingertips on the wall for balance whilst performing heel rises with their knee straight. The number of actual rises performed was counted and compared with the non-injured side.

Complications including re-rupture, iatrogenic sural nerve injury, infection and wound break down rates in addition to symptomatic deep venous thrombosis were compared between groups. Patients were assessed for dysaesthesia in the sural nerve distribution at presentation and following operative repair to assess an iatrogenic sural nerve injury.

### Statistical analysis

All data were analysed using IBM SPSS Statistics Version 25 (IBM Corp, Armonk NY, USA). Descriptive statistics were reported using median (range) and mean ± standard deviation (SD). Data from a prior study reported by our research group [5] was used a priori to estimate sample size. This study utilized similar outcome measures in groups comparing two surgical techniques. Using ATRA as the main outcome with an effect size of 1.4 and alpha value of 0.05, it was estimated that nine individuals per group would be required to adequately power the study [10]. The outcome measures were assessed for significance using a paired samples *t* test. A level of significance was set at *p* < 0.05.

## Results

**Fig. 2:**
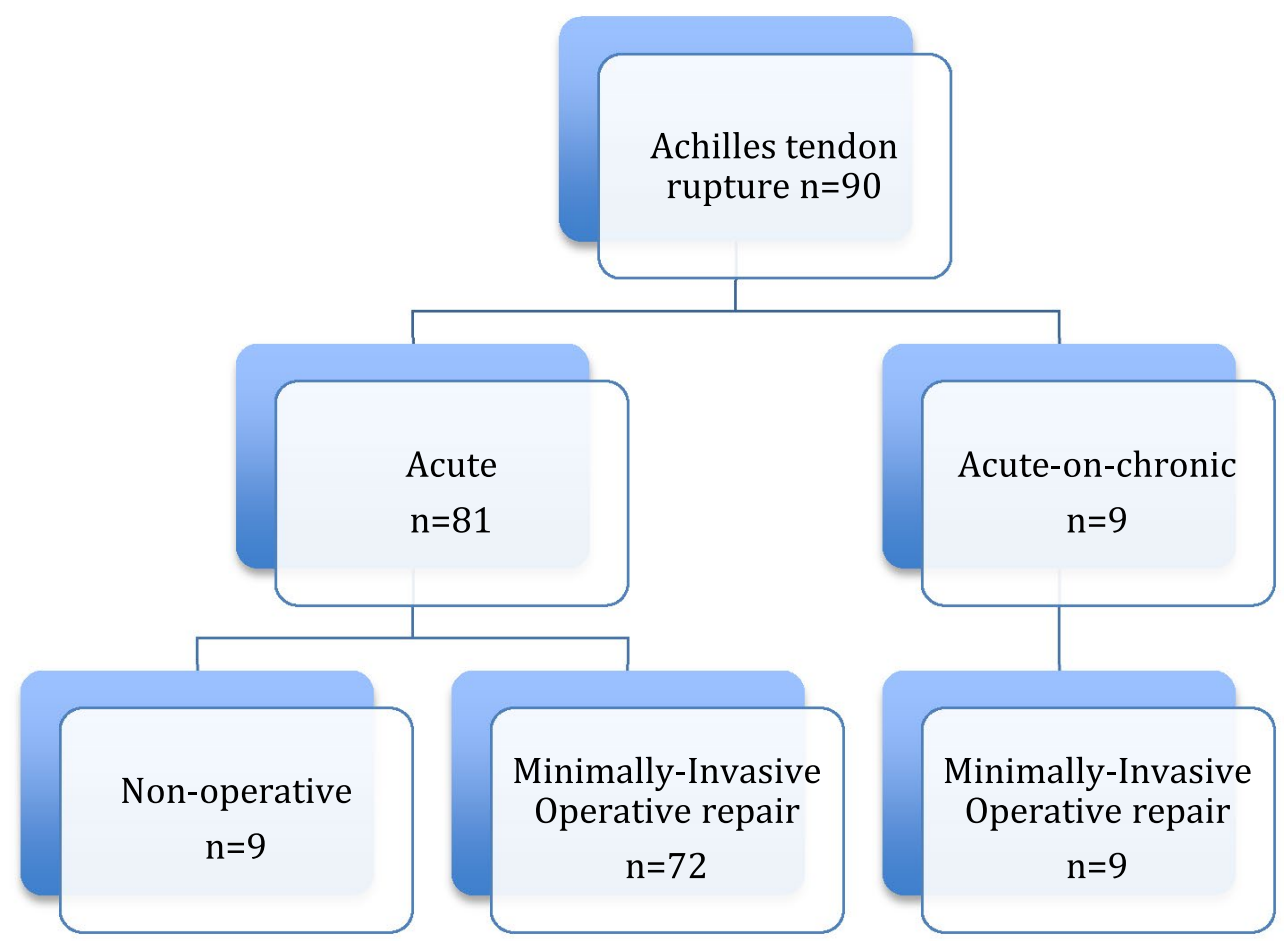
Flow chart of presentations and management of patients following Achilles tendon rupture

From 2014 to 2017, 90 patients presented following Achilles tendon rupture (Fig. 2). Of these, nine patients presented late after 2 weeks following injury receiving no treatment during this period. The rate of delayed presentation was estimated to be 1 in 10 patients. Three other patients presented acutely and received treatment with a below knee back slab in plantar flexion but had operative repair at over 2 weeks following injury. The reasons for the delay in repair included a change in management according to patient preference (*n* = 2) and a week of anticoagulation following the pre-operative recognition of a deep venous thrombosis despite prophylactic low-molecular weight heparin (Fig. 2). One patient presented between 4 and 6 weeks following rupture.

Elapsed time from injury to presentation and the commencement of treatment in the delayed presentation group was 21.8 days (14.9) (range 14–42). Operative repair was performed at 3.4 (2.7) days following the commencement of treatment. These patients were compared with an age-matched cohort (*n* = 9) who presented early, receiving management 0.66 (1.7) (0–5) days following injury and an acute repair 4.44 (2.6) days. There were no differences between age, body weight and body mass index (BMI) between the two groups (Table 1). In the delayed presentation group, four patients sustained their injury participating in activities of daily living, compared to two in the acute group. The remainder sustained their injuries during sports participation; the most common in both groups was football.

**Fig. 3:**
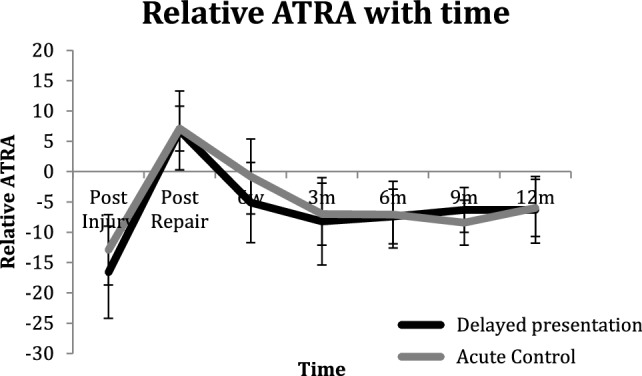
The relative Achilles tendon resting angle (ATRA) with time

**Fig. 4:**
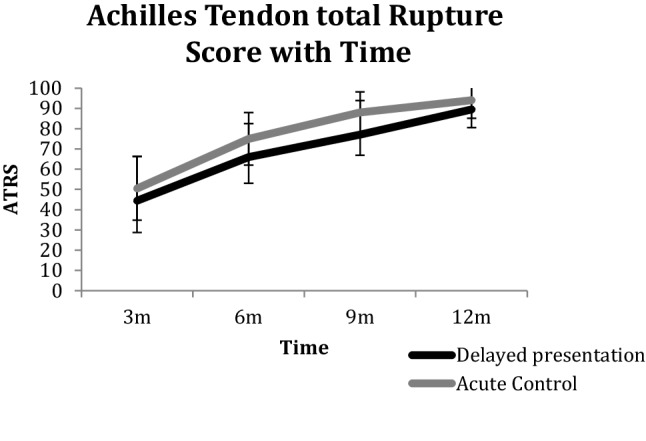
The Achilles tendon Total Rupture Score (ATRS) with time

**Fig. 5:**
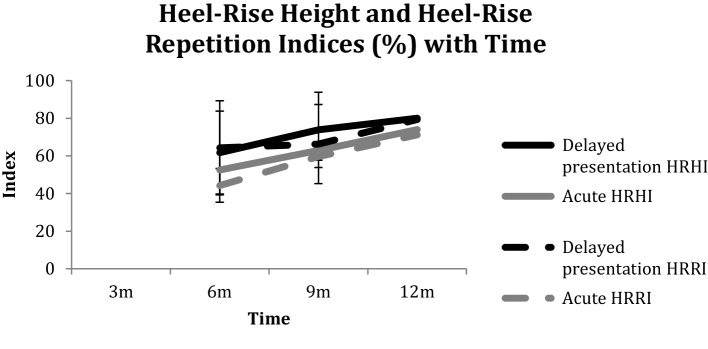
Heel-Rise Height Index (HRHI) and Heel-Rise Repetition Index (HRRI) with time

At 12 months following delayed presentation repair, patients had a relative ATRA of − 6.9° (5.5) compared with − 6° (4.7) (n.s.) (Fig. 3), reported a median ATRS score of 89 (79–99) compared with the acute repair group of 94 (79–100) (n.s.) (Fig. 4), a HRHI of 79% (20) compared with 74% (14) (n.s.) and a HRRI of 77% (20) compared with 71% (20) (n.s.) (Fig. 5). The effect size in terms of the ATRA was found to be 0.176.

At the 3-month time point, five (56%) patients in the delayed presentation group were able to perform a single heel rise compared to two (22%) in the acute group. There was no difference in HRHI or HRRI at the 12-month time point with a HRHI of 81% achieved in the delayed presentation group (Fig. 5, Supplementary material Table 1). The effect size of HRHI and HRRI was found to be 0.29 and 0.3, respectively. Return to sports activity after 12 months of rehabilitation following repair is shown in Table 2.

**Table 2:** Return to sports activity after 12 months of rehabilitation following repair

Return to sports at 12 months	Delayed Mean (SD), median (range)	Acute Mean (SD), median (range)
Tegner	5.1 (2.2), 5 (2–9)	5.6 (1.3), 5 (4–8)
Halasi	5.3 (2.5), 5 (1–9)	5.7 (1.7), 5 (4–9)
Physical Activity Score	4.3 (0.9), 4.5 (3–6)	4.5 (0.9), 4.5 (3–6)
Change in Tegner level/number of patients		
Reduced	5	5
Same	3	3
Improved	1	1
Patient’s perception of performance		
Same or improved	78% (7)	56% (5)

In the delayed presentation group, the mean incision size of the repair site was 4.2 cm (0.67), (3–5 cm). There was one patient who suffered an iatrogenic sural nerve injury (11%) and two patients (22%) who suffered from wound infection requiring antibiotic therapy. One patient required suture removal at 6 weeks post-operatively and at this point the tendon had healed. In the acute group, one patient had symptomatic deep venous thrombosis. There were no cases of re-rupture in either group.

## Discussion

The most important finding of this study is that there were comparable outcomes between patients who received minimally invasive repair following delayed presentation compared with those who presented acutely. Once learned, this technique is a safe method of apposing tendon ends and providing an augmented end-to-end repair through a small incision for delayed presentation [11].

Concerns in terms of the strength of percutaneous relative to open suture configurations have been reported [20, 27]. Although the use of minimally invasive repair techniques for patients presenting after the acute phase following injury presents a potentially attractive option, only one series by Anathattee et al. has been reported [1]. The Achillon jig (Integra, Plainsboro, NJ, USA) has been used to perform operative repair, using a box suture, at 11–31 days following rupture. Patients had ATRS of 91 points at 71 months following injury with no difference in reported limitation score compared to those having a more acute repair [1]. Percutaneous suture configurations including the box suture are weaker than open configurations on biomechanical testing [7, 27] leading to increased ankle dorsiflexion [15], however, good clinical outcomes are reported in acute repair using modified Bunnell and Kessler configurations [4–6]. The use of a minimally invasive repair permits the augmentation of the core suture with a peripheral epitenon running suture to increase the strength of the repair [14, 20].

The patient-reported outcome scores in this series are similar to individuals with chronic rupture and reconstruction previously reported. Becher et al. reported an ATRS of 75 at 5.6 years follow-up following reconstruction, using various techniques, of chronic rupture (> 4 weeks) [2]. Anathattee et al. reported on outcome at 71 months following operative repair performed 11–31 days following rupture, with those patients having an ATRS of 91 points with no difference compared with those having a repair within 10 days of injury [1]. Longitudinally evaluated cohorts in Sweden and Denmark have shown increase in ATRS over time of 7 points from 1 to 7 years and 6.5 points from 1 to 4.9 years, respectively [3, 19]. In this series, it would be reasonable to expect the ATRS to increase further with rehabilitation.

There appeared to be only minor differences in the pattern of alteration in the ATRA with time. The increase in the relative ATRA, however, appeared at an earlier time point in the delayed presentation group exceeding the resting angle of the non-injured side by the 6 weeks point despite protective anterior shell use. The acute group attaining a relatively plantar flexed position at the 3 months evaluation is in agreement with series evaluating the ATRA over time [5, 6]. From operative repair until the 6-week time point, patients were permitted to bear weight on their metatarsal heads using crutches. The increase in ATRA occurred in spite of the theoretically greater strength of repair configuration provided by the circumferential suture. This change in ATRA may relate to either the biology of healing of the repaired tendon following delayed presentation, and/or the weight-bearing technique performed. It is possible that patients with delayed presentation are less attentive to their injury and were less compliant with the metatarsal head weight bearing rather than heel weight-bearing instructions, however, compliance was not assessed. Attempting to walk with a normal gait pattern, whilst wearing a brace, potentially places increased loading on the Achilles tendon, making the tendon more prone to elongation [12]. Once the tendon elongates during the rehabilitation process, this does not fully reverse with time.

Patients in the delayed presentation group had a HRHI of 81% and a HRRI of 77% at the 12 months evaluation point indicating an approximately 20% loss of calf muscle performance. In Becher et al’s series, there was no difference in a heel-rise test height compared with the non-injured side with acute repairs; side difference was mean (SD) 2.9 ± 2.0 cm versus chronic 2.9 ± 2.9 cm [2].

The minimally invasive repair technique used for delayed presentation has a learning curve. The incision was minimized, 4.2 cm (0.67), (3–5 cm), and was centered at the proximal end of the tendon. The incision length is a balance between the length required to insert a suture configuration of the surgeon’s preference, adequate mobilisation of the tendon ends and the risks of increased wound length. A large incision may be more prone to infection and wound breakdown, however, an inadequate incision may also place the patient at risk of these problems because of excessive retraction, bruising and vascular occlusion at the wound edges. Following acute rupture, the tendon ends are still mobile permitting a smaller incision to be used. Tejwani et al. compared the incision length for percutaneous and open repair for acute rupture with 2.5 cm long incisions in the percutaneous group and 7.2 cm incisions for the open group [28].

In delayed presentations, healing occurs with the separated tendon ends adherent to the paratenon resulting in a gap. In chronic cases, the tendon may have to be released using sharp dissection, but in the 4 weeks following delayed presentation dissection may be performed using a blunt instrument [18]. Although blunt instruments are recommended, care must be taken as the paratenon was found to be thin and easily penetrated away from the thickened healing zone. This resulted in the iatrogenic nerve injury in the second case of the delayed presentation group. Sural nerve injury occurred at a high level (14%) in Anathattee et al’s. series, in addition two patients sustained re-rupture [1].

Since a larger incision of mean 4.2 cm was used in the delayed presentation to mobilise adherent separated tendon ends, the incision provided the opportunity to strengthen the core suture repair. Recent meta-analyses of biomechanical studies have shown comparable strength for the Krackow suture and Bunnell suture [30], however, to optimise the strength of the Krackow suture, the locking loops must be inserted beyond the frayed ends of the ruptured tendon [13] increasing incision length. Using this described technique once the adherent tendon ends have been mobilised, and the percutaneous modified Bunnell and Kessler core sutures inserted, the repair strength was also be augmented by a circumferential running [20] or locking suture [14] without increasing the incision length and increasing the associated risks.

Limitations of this study include the small cohort size together with the comparison cohort group used. Only those patients with 2 weeks of delayed presentation and no management during this time period were included. The rate of delayed presentation was estimated to be 1 in 10, lower than that reported elsewhere in the literature [17, 21]. The small number of patients also includes the learning curve of the Julien mobilisation technique and as result the presented complication rate may be disproportionately high. Another limitation is the absence of ultrasonography to confirm gap size. The requirement for minimally invasive repair to release adherent tendon ends to permit end-to-end apposition under direct vision, meant that imaging was not required.

The use of a group of patients presenting acutely as a comparison cohort is also a limitation of this study. An ideal comparison cohort would be a standard open repair using a Krackow suture augmented by the peripheral circumferential suture; however, this method has not been performed for patients at this unit. Prior to commencing the minimally invasive technique, the author’s standard technique for chronic presentations was an open repair augmented with hamstring. For acute repairs, comparison studies have shown no difference in outcomes between end-to-end and augmented repairs [33] with the message that more is not necessarily better [8]. Patients presenting acutely repaired using an identical core suture technique were used as the best available comparison group permitting age matching. The selection of these patients also introduces an element of bias although the first patient of comparable age was chosen to minimise this.

This study shows that the minimally invasive method used to repair the tendon after delayed presentation does produce satisfactory results.

## Conclusions

Patients with delayed presentation following Achilles tendon rupture achieved similar long-term function as those managed acutely with minimally invasive repair consisting of a modified Bunnell and Kessler core sutures with circumferential suture augmentation.

## Electronic supplementary material

Below is the link to the electronic supplementary material.

Supplementary material 1 (DOCX 11 KB)
