# Supplementary material for: No difference in strength and clinical outcome between early and late repair after Achilles tendon rupture
Source: Knee Surg Sports Traumatol Arthrosc. 2018 Dec 29;28(5):1587–94. doi: 10.1007/s00167-018-5340-5 (PMC7176605; doi:10.1007/s00167-018-5340-5)
Supplement: Supplementary file 1 — Supplementary material 1 (DOCX 11 KB) [file 167_2018_5340_MOESM1_ESM.docx]

**Table 1** The Achilles tendon Total Rupture Score (ATRS), Heel-Rise Height Index (HRHI) and Heel-Rise Repetition Index (HRRI) with Time.

|  | Median (range)  Mean (SD) | 3 months | 6 months | 9 months | 12 months |
| --- | --- | --- | --- | --- | --- |
| ATRS | Delayed presentation | 45 (36-100)  53 (21.9) | 66(42-91)  68 (16.5) | 77 (46-92)  74 (16.8) | 90 (69-99)  85 (12.2) |
|  | Acute Control | 51 (39-84)  55 (15.7) | 75 (60-94)  76 (13) | 88 (72-99)  87 (10.1) | 94 (75-100)  91 (8.9) |
|  | p value | n.s. | n.s. | n.s. | n.s. |
| HRHI | Delayed presentation |  | 62 (21) | 74 (22) | 81 (20) |
|  | Acute Control |  | 52 (18) | 63 (20) | 74 (14) |
|  | p-value |  | n.s. | n.s. | n.s. |
| HRRI | Delayed presentation |  | 64 (25) | 66 (25) | 77 (21) |
|  | Acute Control |  | 44 (6) | 60 (9) | 71 (20) |
|  | p-value |  | n.s. | n.s. | n.s. |
